# Sphingolipids as Emerging Mediators in Retina Degeneration

**DOI:** 10.3389/fncel.2019.00246

**Published:** 2019-06-11

**Authors:** M. Victoria Simón, Facundo H. Prado Spalm, Marcela S. Vera, Nora P. Rotstein

**Affiliations:** Instituto de Investigaciones Bioquímicas de Bahía Blanca (INIBIBB), Departamento De Biología, Bioquímica y Farmacia, Universidad Nacional del Sur (UNS), Argentine National Research Council (CONICET), Bahía Blanca, Argentina

**Keywords:** ceramide, sphingosine-1-phosphate, sphingosine, ceramide-1-phosphate, photoreceptor, glia, pigmented epithelium

## Abstract

The sphingolipids ceramide (Cer), sphingosine-1-phosphate (S1P), sphingosine (Sph), and ceramide-1-phosphate (C1P) are key signaling molecules that regulate major cellular functions. Their roles in the retina have gained increasing attention during the last decade since they emerge as mediators of proliferation, survival, migration, neovascularization, inflammation and death in retina cells. As exacerbation of these processes is central to retina degenerative diseases, they appear as crucial players in their progression. This review analyzes the functions of these sphingolipids in retina cell types and their possible pathological roles. Cer appears as a key arbitrator in diverse retinal pathologies; it promotes inflammation in endothelial and retina pigment epithelium (RPE) cells and its increase is a common feature in photoreceptor death *in vitro* and in animal models of retina degeneration; noteworthy, inhibiting Cer synthesis preserves photoreceptor viability and functionality. In turn, S1P acts as a double edge sword in the retina. It is essential for retina development, promoting the survival of photoreceptors and ganglion cells and regulating proliferation and differentiation of photoreceptor progenitors. However, S1P has also deleterious effects, stimulating migration of Müller glial cells, angiogenesis and fibrosis, contributing to the inflammatory scenario of proliferative retinopathies and age related macular degeneration (AMD). C1P, as S1P, promotes photoreceptor survival and differentiation. Collectively, the expanding role for these sphingolipids in the regulation of critical processes in retina cell types and in their dysregulation in retina degenerations makes them attractive targets for treating these diseases.

## Introduction

**FIGURE 1:**
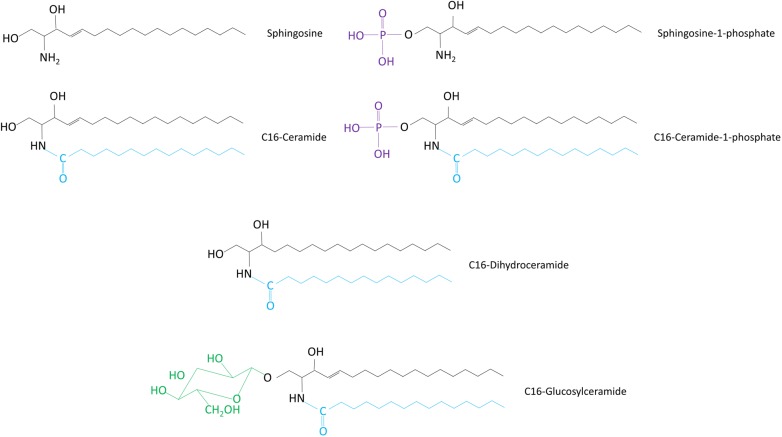
Chemical structures of sphingolipid molecules. The structures of different sphingolipid types are shown. All sphingolipids share a sphingosine backbone (black). This sphingoid backbone is amide-linked to a fatty acid moiety (blue).

Sphingolipids entered the group of Bioactive Lipids about three decades ago; however, they are still regarded by many as newcomers, less familiar than the phosphatidylinositol phosphates, prostaglandins or leukotrienes. Yet, the overwhelming amount of literature accumulated evidences that simple sphingolipids such as Cer, Sph, and their phosphorylated derivatives, S1P and C1P (Figure 1) have taken centre stage in controlling normal and pathological cellular processes throughout the organism.

First identified in the brain by Thudichum in the late 19th century, for virtually another century their functions remained as enigmatic as the Sphinx their name honors. Being lipid molecules, they were considered as ubiquitous membrane components both in animal and plant cells for several decades. Even just as such, they have multiple and relevant roles. They are crucial constituents of lipid rafts and regulate their formation and expansion, essential for building signaling platforms (Grassme et al., 2001); they are also critical for receptor function, membrane conductance and cell–cell interactions, and play key roles in pathogen internalization (Huwiler et al., 2000; Gulbins and Kolesnick, 2003; Hannun and Luberto, 2004). The pioneer work from both Hannun and Kolesnick laboratories three decades ago added an additional dimension to their biological relevance; the groundbreaking findings that Sph inhibits protein kinase C and Cer is a “potential second messenger” in the signaling cascade activated by TNF-α provided the first data as to their role as bioactive lipids (Hannun et al., 1986; Kolesnick, 1987; Dressler et al., 1992; Obeid et al., 1993). Sph and Cer were then established to inhibit cell growth and promote cell death upon different cell stressors. The family of bioactive sphingolipids gained in complexity when their phosphorylated counterparts, S1P and C1P were shown to control the opposing processes, proliferation and cell survival. The sphingolipid family also includes GlucoCer, lactosylceramide, several gangliosides and DHCer.

These bioactive lipids respond to a diversity of cell stimuli by modifying their intracellular levels, thus regulating multiple signaling pathways that finally induce major changes in cell fate. Their roles have strikingly extended to include virtually every aspect of cell biology, from cell cycle, differentiation, endosome and exosome formation, adhesion and migration to angiogenesis, immune response, inflammation and cell death, including autophagy and apoptosis. Recent advances have revealed that sphingolipid mediators play important roles in human disease.

This Review focuses on the functions played by simple sphingolipids in the retina during development and particularly in the onset of retina pathologies. Lipids have long been known to be essential for maintaining both the structure and functionality of the visual system. Docosahexaenoic acid (DHA) has been shown to promote photoreceptor survival and differentiation, and docosanoids, such as Neuroprotectin D1, and elovanoids have relevant neuroprotective roles in the retina (Rotstein et al., 1996, 1997; Jun et al., 2017; Bazan, 2018). Conversely, mutations of lipid metabolizing proteins and chronic misregulation of retinal lipid metabolism have been linked to retinal degeneration (Fliesler and Bretillon, 2010; Friedman et al., 2010; Yu et al., 2011). During the last decade, understanding on the relevance of lipids in the retina has expanded to reveal the involvement of sphingolipids in numerous ocular diseases. Here, we briefly present the structural characteristics and metabolism of sphingolipids and a concise description of the physiological and pathophysiological roles of simple sphingolipids as Cer, Sph, S1P, C1P, and DHCer. We then focus on the information that points to both their deleterious and protective functions in the retina and in retina pathologies, supporting the emerging roles of sphingolipids as novel mediators in retina degenerative diseases.

### Structure and Metabolism of Sphingolipids

The sphingolipid family encompasses a huge diversity of molecular species that place them among the major lipid classes in eukaryotic cells. Sph, a straight, long chain (18–20 carbon atoms) aminoalcohol, is the common backbone shared by all these species (Figure 1). Attachment of a fatty acid to Sph through an amide bond gives rise to Cer, the central molecule in sphingolipid metabolism (Fahy et al., 2005). Further attachment of hundreds of diverse headgroups at the C-1 position of Cer originates the more complex sphingolipids (Figure 1). Taking into account than in addition to these huge number of headgroups, sphingolipids are formed by at least sixty different long-chain bases and dozens of fatty acids varying from twelve to over thirty carbons in length, the number of sphingolipid molecular species is likely in the tens of thousands (Merrill et al., 1993). If we add this up to their complex metabolism, and their ability to interconvert upon different and even opposing cell stimuli, we can start to grasp their extraordinary flexibility for modulating an ample range of cell responses.

**FIGURE 2:**
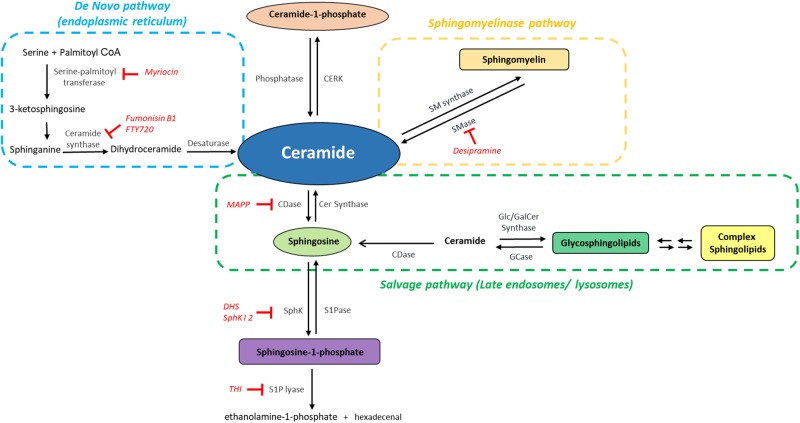
The sphingolipid network: metabolic interconnection between bioactive sphingolipids. Ceramide, the central hub of sphingolipid metabolism, is synthesized by the *de novo* pathway (light blue), from serine and palmitoyl CoA, by the sphingomyelinase pathway, i.e., through hydrolysis of sphingomyelin mediated by sphingomyelinases (SMase) (orange) or by the salvage pathway (green). Ceramide can then be phosphorylated to generate Ceramide-1-phosphate and/or deacylated to form sphingosine, which is then phosphorylated to generate sphingosine-1-phosphate (S1P). The catabolism of S1P mediated by S1P lyase provides the only exit route from the sphingolipid network. CDase, ceramidase; CERK, ceramide kinase; GCase, glucosylceramidase; SMase, sphingomyelinase; SM synthase, sphingomyelin synthase; SphK, sphingosine kinase; SPPase, sphingosine phosphate phosphatase. The inhibitors mentioned in this Review are indicated in red.

A key to the multiplicity and diversity of the signaling roles of sphingolipids is in their intricate and highly interconnected metabolism, their constant cross-conversions that modify their levels upon changes in the environment (Figure 2). Cer is the central hub among these metabolic pathways and can be synthesized by different pathways, *de novo* synthesis, degradation of sphingomyelin (*sphingomyelinase pathway*) and recycling of Sph and complex sphingolipids (*salvage pathway*). *De novo* synthesis begins in the ER (Mandon et al., 1992) with the condensation of L-serine and palmitoyl CoA, catalyzed by SPT; the resulting 3-ketosphinganine is reduced to sphinganine, which is amino-acylated with a chain of 14 to 32 carbons to form diverse DHCer species; finally, the insertion of a *trans* double bond at the C4 position of the sphingoid base backbone by DHCer desaturase gives rise to Cer. SPT, a heteromeric complex, is responsible for opening the entrance to the sphingolipid network. Interestingly, recent evidence has uncovered that subunit mutations causing hereditary sensory and autonomic neuropathy type 1 (HSAN1) shift SPT preference to use alanine and glycine instead of serine (Penno et al., 2010; Bode et al., 2016). This gives rise to a class of atypical 1-deoxysphingolipids, such as deoxy(dihydro)ceramides and 1-deoxysphingosine, shown to induce cell death in various cell types. When elevated, as in HSAN1, they are neurotoxic and contribute to sensory and autonomic neuropathies affecting both cytoskeletal stability, NMDA receptor signaling and membrane properties (Jiménez-Rojo et al., 2014; Güntert et al., 2016). SPT can also change its selectivity for palmitate, using myristate or stearate as substrates (Hornemann et al., 2009; Harmon et al., 2013), further increasing the diversity of sphingolipid molecules.

The newly synthesized Cer can be glycosylated by GlucoCer synthase on the cytoplasmic surface of the Golgi, to render GlucoCer, the precursor of glycosphingolipids, or galactosylated by galactosyl Ceramide synthase in the ER (Figure 2; Raas-Rothschild et al., 2004). It can also receive a phosphocholine head group from phosphatidylcholine and thus generate sphingomyelin (SM), a reaction mediated by SM synthases (Tafesse et al., 2006). In turn, these complex sphingolipids can generate Cer through basal or signal-mediated catabolic pathways. The hydrolysis of the phosphodiester bonds in SM, catalyzed by at least five different SMases, renders Cer through the so-called *sphingomyelinase pathway* (Figure 2). These enzymes present several isoforms differing in subcellular localization, optimal pH range and cation dependence. A prominent example is neutral SMase; a Mg^2+^ -dependent form is localized in the plasma membrane whereas a cation-independent form is found in cytosol (Marchesini and Hannun, 2004); a mitochondrial neutral SMase has also been identified (Wu et al., 2010; Rajagopalan et al., 2015). The acid SMase gene can also generate, through differential trafficking, a cation-independent acid SMase, found in the endosomal-lysosomal compartment and an acid SMase that is secreted extracellularly and is responsible for hydrolyzing SM in the outer leaflet of the plasma membrane in addition to that present in plasma lipoproteins (Jenkins et al., 2009). Activation of SMases in response to diverse stimuli in different compartments provides the means for a rapid Cer generation, crucial for signal transduction.

A third pathway for Cer generation relies on the breakdown of complex sphingolipids in the lysosomal or late endosomal compartment through the reverse activity of different hydrolases, such as specific β-glucosidases and galactosidases, to form Cer, which cannot be released from this compartment. The subsequent activity of at least five different ceramidases generates Sph and its recycling in the ER and reacylation by CerSs yields Cer; this “salvage pathway” (Figure 2) is involved in inflammatory processes (Kitatani et al., 2008; Canals et al., 2018). Finally, exogenous Cer can also be recycled and generate endogenous Cer by the reverse action of ceramidases (Kitatani et al., 2008; Novgorodov et al., 2011).

Cer is phosphorylated by a specific kinase, CerK to form C1P (Wijesinghe et al., 2005). As stated above, deacylation of Cer by ceramidases renders Sph, the phosphorylation of which by one of the two existing SphKs, SphK1 and SphK2, generates S1P (Hait et al., 2006; Figure 2). The irreversible degradation of S1P by S1P lyase, at the cytoplasmic side of the ER, yields ethanolamine-1-phosphate and hexadecenal, providing the only release gate from the complex sphingolipid metabolic cycle (Bandhuvula and Saba, 2007). Comprehensive and detailed accounts of sphingolipid molecular diversity and metabolism can be found in excellent previous reviews (Lahiri and Futerman, 2007; Kitatani et al., 2008; Hannun and Obeid, 2011; Canals et al., 2018).

### To Be or Not to Be…, a Decision for the Sphingolipid Rheostat

By 1995, several seminal findings evidenced that Cer and S1P had opposing cellular roles; whereas growth and survival factors increase S1P levels to stimulate proliferation and survival, different cell stressors promote an intracellular accumulation of Cer, which arrests the cell cycle or induces cell death. The ready interconversion of S1P, Sph, and Cer (Figure 2), prompted by numerous cues that stimulate or inhibit the activities of the enzymes involved, provides a significant tool for determining the patterns of intracellular signaling and deciding the physiological outcome. This understanding led to the proposal that the opposite functions of S1P and Cer transform their signal-mediated interconversion in a sensor of intracellular conditions, and the consequent rapid alteration of the balance between their levels in a key switch in the control of cell fate, later termed as the “sphingolipid rheostat” (Gómez-Muñoz et al., 1995; Cuvillier et al., 1996).

Since then, the collective effort of numerous laboratories has shed new light on the molecular actors and the signaling pathways involved in the intricate cross-reactions of these sphingolipids, included new sphingolipid molecules such as C1P and DHCer, and shown that this rheostat is involved in the induction of multiple pathologies, including neurodegeneration (Taha et al., 2006; Hannun and Obeid, 2008; Young et al., 2013; Newton et al., 2015; Wang and Bieberich, 2018). The extensive available data provides us with an optimal position to complete the unraveling of the sphingolipid universe. The subcellular compartmentalization of their metabolic pathways has been established and the major enzymes giving rise to this diversity of sphingolipids have been identified and cloned during the past three decades. New evidence suggests that different Cer molecular species, generated by different pathways in different compartments might differ in their intracellular roles (Hannun and Obeid, 2011; Hernández-Corbacho et al., 2015). Hence, the relevance of the sphingolipids involved in the rheostat model extends beyond their relative levels and the tightly regulated enzymes involved in their synthesis and degradation to encompass the innumerable and frequently opposite signaling pathways they control, their site of biosynthesis and their release to the extracellular milieu, to finely tune the sphingolipid rheostat.

### Sphingolipids in Eye Pathology

The last 15 years have seen the buildup of a body of evidence pointing to a role for sphingolipids in normal development and function of the retina and in the pathogenesis of ocular diseases. The first link between alterations in sphingolipid metabolism and eye disease originated from lysosomal storage diseases, collectively denominated sphingolipidoses, which arise in mutations in the enzymes or cofactors involved in sphingolipid degradation. These diseases share an early neurodegeneration and visual impairment, with ocular manifestations including retinal vascular abnormalities, degeneration of ganglion cells, and even blindness (Harcourt and Ashton, 1973; Brownstein et al., 1978; Brownstein et al., 1980; Chen et al., 2014).

In Farber’s disease, Cer accumulation in the retina brings on visual dysfunction, with ganglion cells being the most affected (Zarbin et al., 1988). Brain accumulation of Cer occurs in the juvenile form of Batten disease, in which retina neurodegeneration and blindness are early events (Puranam et al., 1997). These early reports underscored the relevance of sphingolipid metabolism in eye pathogenesis; however, few clues existed on how this accumulation led to ocular manifestations. Thrillingly, work from many laboratories over the last decade has implicated sphingolipids such as Cer, S1P, Sph, and C1P in the progression of ocular diseases, including diabetic retinopathy, retinitis pigmentosa, AMD and other neuronal degenerative diseases. We will first summarize the sphingolipid species present in the eye and then review their roles in retinal cells and in the pathogenesis of these diseases.

### Sphingolipid Presence in the Eye

Due to their high complexity, detailed composition analysis of retina sphingolipids is quite recent. Sphingolipids amount to about 11–13 (mole) % of both rat and bovine retinal lipids (Brush et al., 2010). SM is the most abundant species, amounting to 2.4–2.5% of total retinal lipids, whereas Cer and GlucoCer amount to less than 1%. Retinal sphingolipids have abundant (nearly 90%) of saturated, and particularly very long chain saturated fatty acids, with 18:0 and 16:0 being the most abundant fatty acid species. They contain about 2–3% of DHA, 22:6 n–3, the most abundant PUFA, but no VLCPUFA longer than 24 carbons are present (Brush et al., 2010); this is intriguing since the retina is characterized for the abundance of PUFA and VLCPUFA, which are enriched in sphingolipids in other tissues (Aveldaño and Sprecher, 1987; Oresti et al., 2011). In mice retina, almost 80% of Cer contains 16:0 and 18:0, with 21% having 20:0 or longer chain fatty acids (Fox et al., 2006). Bovine rod OS have lower levels of sphingolipids than the whole retina, varying from 3.4 to 6.4 mole % (Brush et al., 2010). SM and Cer from detergent resistant membranes obtained from rod OS are enriched in saturated fatty acids (Martin et al., 2005).

Diverse studies confirm the earlier observations that the sphingolipid profile is modified in retinal pathologies. Cer mass content decreases in retinas from diabetic mice compared to normal retinas, without modifying its fatty acid composition, with a concomitant increase in GlucoCer, and no changes in Sph and SM (Fox et al., 2006). The relevance of particular Cer molecular species in preserving retinal functions has been emphasized by recent findings from the Busik laboratory, demonstrating that overexpression of elongation of very long-chain fatty acids protein 4 (ELOVL4), whose presence is significantly reduced in the diabetic retina, preserves tight junctions and prevents retinal vascular permeability; this effect is parallel to an increase in the levels of Cer having 16 and 24 carbons, and very long chain fatty acids, which localize in and might stabilize tight junctions (Kady et al., 2018). In a retinitis pigmentosa model, the 23H-1 rat, photoreceptor degeneration starts at the beginning of light responsiveness; Cer, S1P and SM increase in the retina early during degeneration, with a reduction in shorter-chain species and an increase in longer-chain species (Stiles et al., 2016).

The fact that sphingolipids are affected during the course of degeneration intuitively makes them likely candidates to have a role in retinal diseases. A combination of studies *in vivo* and *in vitro* has contributed to our understanding of these roles. *In vivo* studies with animal models have provided comprehension on the biological functions of sphingolipids and allowed to test the physiological effects of modulating its levels. Research *in vitro* has allowed dissecting their specific effects on particular cell types and the biochemical pathways involved in these effects. We will now highlight the findings that support that significance of sphingolipids during normal development and in pathologies affecting the retina.

## Ceramide, a Crucial Executioner in Retinal Degeneration

### Cer Metabolism

Cer has a central role in the sphingolipid family, both structural and metabolic. Consisting in a Sph molecule bound to a fatty acid of 16 to 24 carbons length (Fahy et al., 2005), these long acyl chains confer Cer its high hydrophobic properties; unable to exist in a free-solution form in the cytoplasm, it is the most hydrophobic lipid in biological membranes (Castro et al., 2014). This apparently static, membrane-confined location of Cer does not preclude it from having key intracellular actions, as the regulation of cell cycle and cell death.

As Cer is synthesized in the ER, its hydrophobic nature demands a transport mechanism to reach its diverse cellular destinations. Cer transport to the *trans* Golgi region, to generate SM, involves a CERT, which carries it in a non-vesicular manner (Hanada et al., 2009). Cer has very different patterns of tissue distribution and functions (Bartke and Hannun, 2009; Kurz et al., 2018). Interestingly, six specific types of CerSs (CerS 1–6) have been identified, each of them attaching acylCoAs differing in their chain length to the sphingoid backbone, thus generating different molecular species of Cer, with distinct cellular effects (Levy and Futerman, 2010; Park and Pewzner-Jung, 2013; Wegner et al., 2016). The distribution of these CerSs differs between organs and even between cell types in the same organ, contributing to a characteristic Cer composition (Laviad et al., 2008; Kremser et al., 2013).

### Ceramide Biological Functions

Numerous cellular stressors, such as oxidative stress, absence of trophic factors, chemotherapy, and UV radiation, activate the synthesis of Cer; the consequent increase in Cer levels mediates many cell-stress responses, including the regulation of cell growth, differentiation (Okazaki et al., 1989), senescence (Trayssac et al., 2018), proliferation, necrosis, apoptosis and autophagy (Obeid et al., 1993; Hannun and Obeid, 2008; Scarlatti et al., 2004). These multiple roles of Cer result from its ability to act both at membrane level and as an intracellular messenger. Cer is a key modulator of membrane dynamics; as a cone-shaped lipid, it readily forms non-lamellar phases with increased negative spontaneous curvature, thus promoting membrane invagination, budding and fusion (Holopainen et al., 2000; Stancevic and Kolesnick, 2010). Cer initiates multiple signaling pathways through the rapid formation or expansion of Cer-enriched microdomains in the plasma or outer mitochondrial membrane. These microdomains allow for the interaction and/or oligomerization of different proteins, such as death receptors in the former or Bax in the later that then signal the activation of diverse death programs (Grassmé et al., 2001; Ganesan et al., 2010).

In addition, Cer triggers many of its effects by acting as a lipid second messenger, through the activation of several intracellular targets. Cer activates protein phosphatase PP1A and PP2A (Chalfant et al., 1999) and regulates protein kinase C zeta (PKCζ) and Akt activity (Wang et al., 2005; Canals et al., 2018; Hannun and Obeid, 2018) as well as raf-1 and the kinase-suppressor of Ras, significantly changing the level of phosphorylation of various key substrates (Ruvolo, 2003).

Among the multifaceted roles of Cer, autophagy and cell death have been those that have drawn more attention. Cer controls both autophagy-mediated cell survival and cell death by regulating nutrient transport, ER stress and mitophagy (Dany and Ogretmen, 2015). Still, Cer has been more frequently identified as a key cell death player (Galadari et al., 2015), activating both the intrinsic and extrinsic pathways of apoptosis. Although its actual contribution to the apoptotic response in living cells has been unclear, multiple *in vitro* studies suggest that Cer might initiate cell death by acting directly on mitochondria. In an interesting recent work, a diverting CERT-mediated Cer transport to mitochondria has been shown to trigger Bax-dependent apoptosis (Jain et al., 2017).

Knowledge on the role of Cer in different pathological processes is vast and constantly expanding, and there are excellent reviews covering its functions (Canals et al., 2018; Hannun and Obeid, 2018; Kurz et al., 2018). We will here focus on Cer functions in retinal physiology and pathologies.

### Ceramide in the Retina

#### Cer and Retina Degeneration

Acharya’s group provided the first direct link between Cer and the death of retinal neurons in a *Drosophila* model of retinal degeneration. Using photo-transduction mutants, they demonstrated Cer has a crucial role in photoreceptor fate; keeping Cer levels low by preventing its *de novo* synthesis or by targeting neutral ceramidase suppresses photoreceptor death in the *Drosophila* mutants (Acharya et al., 2003). In functional null mutants of *Drosophila* ceramidase, photoreceptors degenerate in a light-dependent manner, do not respond to light stimulus and have no effective turnover of rhodopsin; in turn, overexpression of ceramidase, even in tissues distant from photoreceptors, suppresses their degeneration in arrestin mutants and facilitates membrane turnover in a rhodopsin null mutant (Acharya et al., 2008). Furthermore, a *Drosophila* mutant in the CerK shows increased Cer levels leading to the loss of phospholipase C activity and inhibition of phototransduction, ultimately accompanied by photoreceptor degeneration (Dasgupta et al., 2009).

Along the last decade, increasing evidence points to a key involvement of Cer in the onset of retina degeneration in mammals (Table 1). The gradual loss of photoreceptors observed in a rabbit model of retinal detachment correlates with the production of Cer (Ranty et al., 2009). Intra-vitreal C2-Cer injection causes vision loss in rats, increasing apoptosis and expression of glial fibrillary acidic protein, a marker of gliosis (Lou et al., 2017). A recent work in a mouse model of Farber disease, with a deficiency in acid ceramidase activity, provides direct evidence of accumulation of Cer in the retina, associated to inflammation and severe visual loss (Yu et al., 2018). Ischemia increases the expression of acid SMase, increasing Cer levels and leading to retinal degeneration in wild type mice; reduction of this expression in an acid SMase +/- mouse model decreases Cer levels, protects retina structure and preserves its function after ischemic injury (Fan et al., 2016). However, the maintenance of a basal acid SMase activity is necessary for preserving normal retina structure and function; a total deficiency in acid SMase, as occurs in acid SMase knockout mice, leads to the disruption in lysosomal function and prominent photoreceptor degeneration (Wu et al., 2015). Mandalt’s group showed alterations in the sphingolipid profile in P23H-1 rat retinas during degeneration, with an increase in neutral SMase activity leading to higher Cer levels at PN22. Treatment with FTY720, an established CerS inhibitor, partly decreases neutral SMase activity and delays the alterations in retina structure and functionality, granting partial neuronal protection. However, this treatment also leads to a stimulation of acid SMase activity and the consequent increase in Cer levels, which might explain why FTY720 does not fully prevent degeneration (Stiles et al., 2016). This underscores the relevance of establishing not only the pathways leading to Cer increase in each retinopathy but also whether inhibition of a major pathway promotes the activation of further biosynthetic paths, to identify the targets to effectively promote photoreceptor survival.

**Table 1:** Ceramide and sphingosine functions in the retina.

Sphingolipid	Retinal Process
Ceramide (Cer)	**Photoreceptor death**
	↓ Cer levels rescues photoreceptors from death in *Drosophila* RP models (Acharya et al., 2003)
	↑ Cer levels induces photoreceptor loss of function and death in *Drosophila* (Dasgupta et al., 2009)
	Photoreceptor death induced by retinal detachment correlated with ↑ Cer levels in rabbits (Ranty et al., 2009)
	↓ Cer levels by Myoricin treatment (*de novo* Cer synthesis inhibitor) rescue photoreceptors in a *rd10* mouse model (Strettoi et al., 2010)
	↓ Cer levels by inhibiting *de novo* synthesis with FTY720 delays retinal degeneration in P23H-1 rats (Stiles et al., 2016)
	↓expression of acid SMase protects the retina and preserves its function after ischemic injury in an acidic SMase +/- mice model (Fan et al., 2016)
	Intra-vitreal C2Cer injection causes vision loss in rats (Lou et al., 2017)
	C2Cer treatment induces photoreceptor and amacrine cell death in rat retinal neuronal cultures (German et al., 2006)
	Oxidative stress ↑ Cer levels and leads to cone cell death in 661W cell line (Sanvicens and Cotter, 2006)
	Brief C2Cer treatment induces photoreceptor death by activating parthanatos in rat retinal cultures (Prado Spalm et al., 2018)
	**Retinal Pigment Epithelium (RPE) dystrophy**
	C2Cer induces caspase-dependent death in rat RPE cells (Tomita et al., 2000)
	Oxidative stress ↑ Cer levels and activates cell death in ARPE-19 cells (Sugano et al., 2018)
	C2Cer treatment induces apoptosis in non-polarized, but not in polarized RPE cells (Zhu et al., 2010)

Sphingosine (Sph)	**Photoreceptor death**
	Oxidative stress ↑ Sph synthesis, preceding the onset of photoreceptor death *in vitro* (Abrahan et al., 2010)
	Addition of exogenous Sph induces mitochondrial-dependent photoreceptor death (Abrahan et al., 2010)
	**RPE alterations**
	Increased levels of Sph in SMase knockout mice match with age-dependent retina degeneration and RPE alterations, and an impaired autophagic flux (Wu et al., 2015)
	Overexpression of acid ceramidase in ARPE19 cells ↑ Sph levels and protects these cells from oxidative stress (Sugano et al., 2018).


Diabetic retinopathy is the major cause of blindness among working age adults; increased Cer levels have been associated to reduction of insulin action (Chaurasia and Summers, 2015) and recent research establishes Cer as a relevant player in the progression of this retinopathy. Acid SMase is highly activated in the diabetic retina, particularly in retinal endothelium, elevating Cer levels and contributing to the pro-inflammatory changes in this tissue; noteworthy, DHA, which is decreased in the diabetic retina (Tikhonenko et al., 2010), downregulates the expression of acid SMase in human retinal endothelial cells (Opreanu et al., 2010; Busik et al., 2012; Hammer and Busik, 2017). Acid SMase vascular isoform specifically increases in the retinas of diabetic animal models at the vasodegenerative stage, whereas its absence or downregulation with DHA prevents capillary formation and cytokine production (Opreanu et al., 2010, 2011; Busik et al., 2012; Chakravarthy et al., 2016). Activation of acid SMase and the consequent Cer increase appear as relevant contributors to the pathogenesis of diverse retina degenerations, and reestablishing an adequate balance in sphingolipid levels emerges as essential to maintain retina functionality.

#### Ceramide and Photoreceptor Death

**FIGURE 3:**
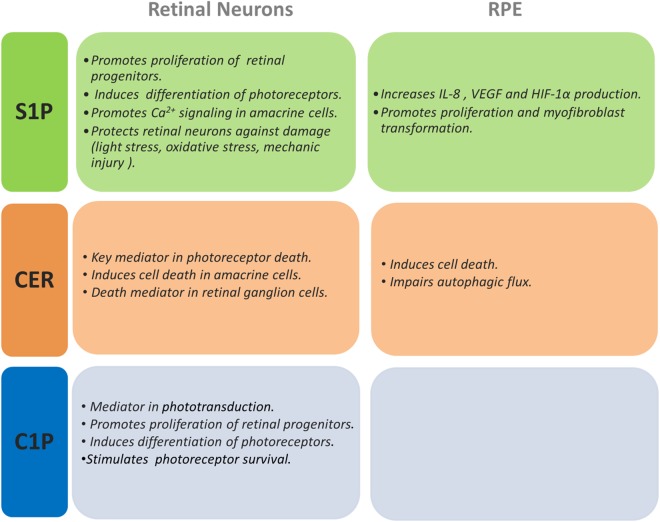
Actions of S1P, Cer, and C1P on retinal neurons and RPE cells. This figure summarizes the effects shown for S1P, Cer, and C1P on different retinal neurons and RPE cells.

**FIGURE 4:**
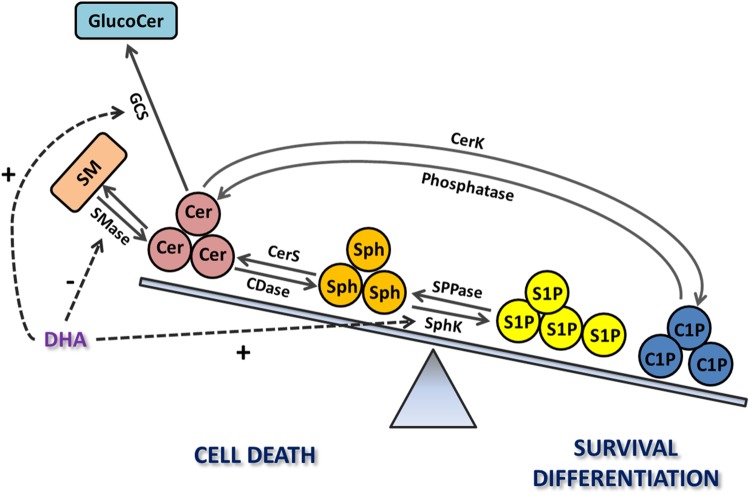
The relevance of the sphingolipid rheostat in retina photoreceptors. Increases in Cer levels through the activation of its *de novo* synthesis or the SMase pathway trigger photoreceptor death; this death is also induced after Cer hydrolysis to increase Sph levels, mediated by ceramidase (CDase). In turn, S1P and C1P promote photoreceptor survival and differentiation. Docosahexaenoic acid (DHA) promotes survival by regulating the sphingolipid rheostat, inhibiting SMase or stimulating the synthesis of glucosylceramide (GlucoCer) to decrease Cer levels or enhancing the synthesis of S1P. Exogenous addition of C1P also promotes photoreceptor survival and differentiation.

The work of several groups, including ours, has provided direct links between Cer increase and the onset of photoreceptor death. This death is the hallmark of most retinal degenerative diseases with very diverse etiologies (Chang et al., 1993; Portera-Cailliau et al., 1994; Sancho-Pelluz et al., 2008); hence, uncovering common molecular mechanisms and mediators is essential to identify new therapeutic targets. *In vitro* studies have been fundamental to establish Cer as a mediator of photoreceptor death. Our group demonstrated that oxidative stress increases the *de novo* synthesis of Cer preceding photoreceptor death in rat retina cultured neurons, whereas inhibiting this synthesis with fumonisin B1, a CerS inhibitor, prevents this death (German et al., 2006). These neurons present low levels of DHA, which has been shown to be neuroprotective for photoreceptors (Rotstein et al., 1996, 1997). DHA supplementation protects photoreceptors from C2-Cer and oxidative stress-induced death, promoting the synthesis of GlucoCer (German et al., 2006). Cotter’s group extended Cer role in oxidative stress-induced death of photoreceptors; they showed that treatment of 661W cells, a cone cell line, with a nitric oxide donor increases Cer levels by activating acid SMase; inhibition of Cer synthesis by an acid SMase inhibitor (Desipramine) prevents cone cell death (Sanvicens and Cotter, 2006). Interestingly, induction of oxidative stress with H_2_O_2_ also raises Cer levels and promotes cell death in 661W cells, whereas inhibiting *de novo* synthesis of Cer with Myriocin, a serine palmitoyl transferase inhibitor, prevents Cer increase preserving cell viability (Fabiani et al., 2017). The buildup of Cer, arising from the activation of different biosynthetic pathways in rod and cone photoreceptors, emerges as a death arbitrator in photoreceptors, suggesting that pharmacological prevention of this increase might have therapeutic potential (Figure 3, 4).

*In vivo* studies have supported this hypothesis. Cer levels increase during the peak of photoreceptor degeneration in the *rd10* mouse, a mouse model of Retinitis Pigmentosa, and treatment with Myriocin noticeably prevents photoreceptor loss and preserves both photoreceptor morphology and retina functionality (Strettoi et al., 2010). Although the genetic mutations in Retinitis pigmentosa and in animal models of this disease affect mostly rods, their death is eventually followed by the death of cones; noteworthy, Myriocin was also effective as a strategy to promote cone survival in the same *rd10* model, even after rod death (Piano et al., 2013).

Increasing evidence suggests that Cer activates death pathways alternative to those of canonical apoptosis to induce photoreceptor demise. Cer increase triggers several pathways in 661W cells subjected to oxidative stress; it not only induces the mitochondrial pathway and subsequent activation of caspases, but also promotes Ca^2+^ increases in both mitochondria and cytosol that precede the activation of calpain-mediated death and activates cathepsin D pathway as well (Sanvicens and Cotter, 2006).

We have recently established that Cer activates PARP-1 to induce photoreceptor death. Cer induces photoreceptor death in cultured rat retina neurons in a caspase-independent process, involving generation of ROS, increase in mitochondrial permeability, activation of PARP-1 and calpain, accumulation of poly ADP-ribose polymers and nuclear translocation of AIF (Prado Spalm et al., 2018), which are features of a recently unveiled death process named Parthanatos (David et al., 2009). Notably, inhibition of both PARP-1 and calpain activity rescues photoreceptors from Cer-induced death (Prado Spalm et al., 2018). Paquet-Durandt’s group established PARP-1 activation as a common non-apoptotic mechanism involved in retinal neurodegeneration in animal models encompassing the major groups of inherited human blindness (Arango-Gonzalez et al., 2014). PARP-1 activity is crucial in the induction of photoreceptor death in the *rd1* an*d rd2* mouse models (Sahaboglu et al., 2017). Activation of PARP-1 has only been associated with Cer-induced death in neuroblastoma cells (Czubowicz and Strosznajder, 2014); hence, these findings not only link for the first time Parthanatos with Cer-induced photoreceptor death but support Parthanatos as a novel cell death routine triggered by Cer. Although different retinal neurodegenerative diseases may have particular cell death mechanisms, taking together these studies Parthanatos appears as a central, shared photoreceptor death-process with Cer as a crucial mediator in these degenerations. Targeting Cer overproduction or Parthanatos key molecular actors might provide enticing strategies for novel, disease-independent treatments for retina neurodegenerations.

#### Cer as a Death Mediator in Other Neuronal Types in the Retina

Cer role as a mediator in the induction of cell damage is not confined to photoreceptors. A 24 h treatment with C2-Cer induces death of amacrine neurons in retina neuronal cultures (German et al., 2006). Optic nerve crush, which triggers the injury to retinal ganglion cells in the rat retina, increases the expression of enzymes involved in Cer biosynthesis (Agudo-Barriuso et al., 2013). Gene expression profiling in injured retinal ganglion cells following overexpression of the *Sox11 transcription factor* revealed that genes associated with Cer biosynthetic and metabolic processes are upregulated, suggesting the activation of a Cer-induced cell death pathway (Norsworthy et al., 2017).

#### Cer and RPE Degeneration

Cer also plays a pivotal role in diseases affecting RPE function, such as AMD and Stargardt disease. Atrophy of RPE cells followed or accompanied by photoreceptor cell death is usually the final outcome of all forms of retinitis pigmentosa, despite their different etiologies. RPE atrophy, i.e., RPE cell death, in the macular region is the primary event in dry AMD and a common event in early stage wet AMD (Al-Zamil and Yassin, 2017). C2-Cer induces death of human and rat RPE cultured cells, which involves caspase activation and is partially prevented by antioxidants and growth factors (Tomita et al., 2000; Kannan et al., 2004; Sreekumar et al., 2009). Oxidative stress increases Cer levels and induces apoptosis in human RPE cells and this was replicated by using C2- and C6-Cer (Barak et al., 2001). Oxidative stress increases both Cer and hexosyl-Cer levels in ARPE-19 cells, a human RPE cell line, promoting cell death; this death is prevented by over-expression of acid ceramidase, which increases Sph levels but not those of S1P (Sugano et al., 2018). Interestingly, increased serum levels of hexosyl-Cer have been reported in patients with late stage AMD (Pujol-Lereis et al., 2018). Noteworthy, oxidative stress leads to increased S1P levels in control RPE cells, which might reflect an attempt to counteract Cer action toward cell death (Sugano et al., 2018). Laser exposure, a frequent treatment for retina neovascularization, induces human RPE cell death concomitant with Cer overproduction (Barak et al., 2005).

Cer significance as a mediator of RPE cell death is also evidenced by the induction of cell death in ARPE19 cells overexpressing neutral SMase3. Intracellular Cer increase and cell death are proportional to the amount of the transfected enzyme and SMase3 overexpression also inhibits proliferation in ARPE19 cells (Zhu et al., 2010). Intriguingly, C2-Cer treatment induces apoptosis in non-polarized RPE cells but does not affect differentiated and polarized RPE cells (Zhu et al., 2010). This differential susceptibility suggests that while healthy, polarized cells forming the RPE monolayer *in vivo* may be resistant to injures leading to an eventual Cer increase, non-polarized, activated RPE cells, frequent in late AMD lesions and in proliferative vitreoretinopathies, might be more susceptible to increased Cer levels resulting from chronic retina injuries (Figure 3).

Cer increase is common to several diseases affecting RPE cells. Excessive acid SMase activation and the subsequent Cer accumulation have been related to the pathogenic changes of RPE cells in diabetic retinopathy. High glucose enhances acid SMase expression, increasing Cer levels in ARPE19 cells. Interestingly, microRNAs (miR) appear as novel players in the modulation of sphingolipid metabolic enzymes. Work from the Busik laboratory showed miR-15a participates in the regulation of Cer levels; its expression is decreased by high glucose whereas its overexpression downregulates acid SMase expression in human RPE cells, restoring normal Cer levels (Wang et al., 2016). This adds an additional complexity to the intricate regulation of sphingolipid metabolism and underscores the usefulness of its modulation in disease treatment.

Acid SMase activation has been shown in aged RPE and in a Stargardt disease mouse model, and the consequent Cer increase impairs autophagic flux, which is crucial for preserving functional RPE and photoreceptor cells (Toops et al., 2015). Efficient endolysosome function in RPE is essential for its phagocytic role and autophagic clearance of cellular debris, and endolysosomal dysfunction is characteristic of neurodegenerative diseases. Recent findings show acid SMase-derived Cer increase and subsequent endolysosomal dysfunction contribute to internalization of the complement protein C3, abnormal levels of which have been implicated in AMD; inhibiting Cer synthesis with desipramine decreases this dysfunction and C3 internalization (Kaur et al., 2018). Significantly, epidemiological studies indicate that the use of tricyclic antidepressants, like desipramine, is associated with a statistically significant protective effect against developing early AMD (Klein et al., 2001), emphasizing the significance of Cer increase in RPE cell dysfunction and AMD progression (Figure 3).

Several signaling pathways participate in Cer-induced RPE cell death. C2-Cer induces the over-production of ROS, activating the intrinsic apoptotic pathway, with a subsequent increase in mitochondrial membrane permeability and caspase-3 activation (Kannan et al., 2004). Both H_2_O_2_ and UV radiation induce ER and stress-activated protein kinase (AMPK) axis, which promote RPE cell death (Yao et al., 2013). Tunicamycin treatment of cultured RPE cells induces NF-κB nuclear translocation, increases in nitric oxide synthase 2 expression and nitrotyrosine formation leading to cell death, which is prevented with a neutral SMase inhibitor, implying Cer generation is involved in this death (Kucuksayan et al., 2014). These data suggest a key role for ER-stress induced by Cer in RPE cell death and a disease-specific activation of SMases.

Taking together the above studies, Cer distinctly emerges as a common pathological mediator in numerous retinopathies of diverse etiologies, activating multiple downstream pathways that induce both the degeneration of rod and cone photoreceptors and RPE dysfunction and death. Pharmacological interventions to prevent Cer increase might provide useful tools for treating multiple retinopathies, in a disease-independent mode. The preservation of retina morphology and functionality by the inhibition of Cer synthesis (Strettoi et al., 2010; Stiles et al., 2016) sustains this proposal. In addition, DHA provides an exciting “proof of concept,” emerging as a modulator of several enzymes of sphingolipid metabolism in order to prevent Cer accumulation, thus tilting the sphingolipid rheostat toward cell survival (Figure 4).

## Sphingosine, a Deadly Messenger in the Retina?

### Sph Biological Functions

The finding that Sph inhibits protein kinase C was one of the first supporting a role for sphingolipids in cell signaling (Hannun and Bell, 1989; Merrill et al., 1989). Later work demonstrated that in addition to this modulatory function, Sph, as its precursor Cer, is a crucial signal for cell death. Sph levels increase during the early stages of cell death, and its addition can trigger this death (Cuvillier, 2002). Oxidative stress, radiation and chemotherapy enhance Cer and Sph generation promoting senescence, cell cycle arrest or cell death (Ogretmen and Hannun, 2004; Hannun and Obeid, 2018). Although Sph increase might result from a decreased SphK activity, as occurs in radiation-resistant prostate cancer cells (Nava et al., 2000), the rapid deacylation of Cer is the main source of Sph increase. The increase in Cer usually precedes that of Sph, suggesting that Cer hydrolysis by ceramidases gives rise to this increase (Ohta et al., 1995; Cuvillier et al., 2000). Precisely, the fact that Cer can be hydrolyzed to Sph, and Sph reacylated to generate Cer made it difficult initially to discern their effects. However, exogenous Sph induces apoptosis in cell lines such as Jurkat cells and rhabdomyosarcoma cell lines even when its conversion to Cer is inhibited (Cuvillier et al., 2000; Phillips et al., 2007) and Sph has been shown to act independently from Cer and at an earlier step in the apoptotic pathway in human leukemic cells (Sweeney et al., 1996). Conversely, inhibiting the synthesis of Sph reduces the induction of apoptosis by different stimuli (Lépine et al., 2004; Suzuki et al., 2004). Sph is now an established second messenger, rapidly generated by different apoptotic stimuli to induce cell death. Sph signals this death through mitochondrial membrane permeabilization, formation of ROS, cytochrome c release, and caspase-3 activation (Sweeney et al., 1996; García-Ruiz et al., 1997; Cuvillier et al., 2000, 2001). Mitochondrial permeabilization is crucial for Sph induction of cell death; Sph induces the downregulation of Bcl-2 levels (Sweeney et al., 1996) whereas Bcl-xl overexpression prevents the onset of apoptosis in spite of the increase in Sph levels (Cuvillier et al., 2001).

### Sph Functions in the Retina

Scarce information exists regarding Sph roles in the eye (Table 1). Initial data in *Drosophila* mutants showed that expression of ceramidase suppresses retinal degeneration in arrestin mutant flies, in parallel to a decrease in Cer levels; however, these mutants show enhanced degeneration of photoreceptors when they are raised with a Sph-enriched diet, suggesting that Sph is not involved in preventing degeneration (Acharya et al., 2003).

Sph functions in mammalian retina are still ill-defined. Sph induces cell death in amacrine and photoreceptor neurons in culture (Abrahan et al., 2010); oxidative stress rapidly enhances Sph synthesis, preceding the onset of photoreceptor death *in vitro*. In turn, inhibition of Sph synthesis by blocking Cer breakdown with an alkaline ceramidase inhibitor, MAPP, markedly decreases oxidative stress-induced photoreceptor death. Moreover, exogenous Sph promotes photoreceptor death, even when Cer synthesis is inhibited, implying that Sph is responsible for this death. As reported in different cell types, Sph promotes apoptotic death by inducing mitochondrial permeabilization in photoreceptors, increasing ROS formation and cytochrome c release, whereas inhibiting Sph synthesis prevents mitochondrial permeabilization (Abrahan et al., 2010).

As occurs with Cer (German et al., 2006), DHA prevents Sph-induced apoptosis, enhancing the expression of SphK1 and its translocation to the plasma membrane (Miranda et al., 2009; Abrahan et al., 2010). Noteworthy, inhibition of SphK1 activity with DHS blocks DHA protection, implying that the decrease in the levels of Sph and/or the generation of S1P are required for DHA protective effect (Abrahan et al., 2010). As a whole, these findings suggest that oxidative stress increases the generation of Cer and Sph in retina photoreceptors, which act as second messengers, inducing mitochondrial dysfunction and increased generation of ROS (Rotstein et al., 2010), to activate cell death. Lowering Sph levels, by preventing its synthesis or promoting its phosphorylation to S1P effectively prevents photoreceptor death, accentuating the relevance of manipulating sphingolipid metabolism in photoreceptor survival.

Whether Sph acts as a deadly messenger promoting degenerative changes in other retinal cell types is still unclear. In acid SMase knockout mice, increased levels of Sph and normal levels of Cer are found in the retina (Wu et al., 2015), probably due to compensatory changes in the activities of other sphingolipid enzymes, aimed at maintaining Cer levels constant. These mice show age-dependent retina degeneration and RPE alterations, with an impaired autophagic flux (Wu et al., 2015); it still remains to be defined whether Sph increase leads to these changes. Puzzlingly, overexpression of acid ceramidase in ARPE19 cells increases their Sph levels, and protects these cells from oxidative stress, with no concomitant increase in S1P levels (Sugano et al., 2018). Hence, much remains to be investigated regarding Sph actions in the retina in order to design new therapies through the adequately modulation of sphingolipid levels.

## Sphingosine-1-Phosphate, a Formidable Double-Edged Sword in the Retina

### S1P Synthesis

Sphingosine-1-phosphate is a crucial lipid intermediate in the complex sphingolipid metabolism. It has a polar head group (phosphate), and a long-chain sphingoid base backbone (Sph) (Saba and Hla, 2004; Figure 1). Sph is phosphorylated to generate S1P by two different SphKs, SphK1 and SphK2 (Maceyka et al., 2005; Figure 2). SphK1 is found mainly in the cytosol, close to the cell membrane, in nearly every cell type. When activated, it translocates to the plasma membrane, where Sph is localized. Its structure, functions and roles in disease are widely identified. Much less is known on SphK2; predominantly localized in nuclei and mitochondria, its expression is tissue and time of development dependent (Hait et al., 2009; Strub et al., 2011). Its structure, functions and the processes in which it is involved are poorly understood. Its most recognized function is its ability to phosphorylate FTY720/Fingolimod, a Sph analog and the first oral pro-drug to be approved for the treatment of multiple sclerosis by the FDA.

### S1P as an Intracellular and an Extracellular Messenger

Notably, in spite of being chemically identical, S1P molecules from nuclei or cytoplasm perform different functions. While nuclear S1P works as a histone deacetylase (HDAC) inhibitor to epigenetically regulate gene transcription (Hait et al., 2009), cytoplasmic S1P acts as a second messenger or as an extracellular ligand. To function as an extracellular ligand, the S1P produced inside the cell is exported by specific transporters, such as Spinster 2 (Spns2) (Osborne et al., 2008; Kawahara et al., 2009; Spiegel et al., 2019) or ABCA1 (Sato et al., 2007), ABCC1 (Mitra et al., 2006), and ABCG2 (Takabe et al., 2010). Once outside the cell, S1P acts in a paracrine or autocrine fashion, a process known as “inside-out signaling” (Takabe et al., 2008). As an extracellular ligand, S1P interacts with five S1P G protein-coupled membrane receptors (S1PRs), termed S1P_1-5_. Most cells express one or more subtypes of S1PRs, and depending on the G-protein they are coupled with, S1PRs exhibit unique properties and regulate different cellular processes. Downstream effectors of S1PRs include adenylate cyclase, PI3-kinase, phospholipase C, protein kinase C and intracellular calcium (Hla et al., 2001; Spiegel and Milstien, 2002). In addition, S1P has been proposed to signal through S1PRs, in a paracrine fashion, upregulating the transcription of SphK1 and thus activating the S1P/SphK1 axis; this “outside-in” signaling pathway has been shown to contribute to the progression of diabetic retinopathy (Huang et al., 2014).

Sphingosine-1-phosphate is a pleiotropic bioactive lipid mediator; its cellular concentration responds to different stimuli and is tightly regulated. Concentrations of S1P in blood and lymph are actually higher than in tissues (Cyster and Schwab, 2012; Olivera et al., 2013; Nagahashi et al., 2016). The final concentration of S1P in any tissue depends on the balance between its synthesis and its degradation, with S1P degrading enzymes playing an important role in maintaining the low tissue levels of S1P. The main enzymes that degrade S1P are S1P lyases and SPP. SPP has two isoforms, SPP1 and SPP2, which catalyze the reversible dephosphorylation of S1P, generating Sph that it is then converted to Cer by CerS (Mandala, 2001; Pyne et al., 2009). S1P lyases are responsible for irreversibly degrading S1P to hexadecenal and ethanolamine-1-phosphate, removing S1P from the sphingolipid metabolic routes and providing the only escape path from this complex metabolism (Bandhuvula and Saba, 2007).

Sphingosine-1-phosphate induces a wide spectrum of cellular effects, including proliferation, differentiation, survival, migration, angiogenesis and immune responses (Tabasinezhad et al., 2013). These pleiotropic effects grant S1P a key role in several diseases, including cancer, inflammation, autoimmunity, atherosclerosis and fibrotic disorders (Knapp, 2011; Maceyka et al., 2012; Takuwa et al., 2013). The highly conserved relevance of S1P and its regulation of important biological functions in organisms ranging from yeast and plants to flies and vertebrates highlight its significance in cell signaling.

The role of S1P in immune functions is well demonstrated, being involved in infections, allergy and chronic inflammation (Aoki et al., 2016). Its key role in neuroinflammation is evident in the successful use of FTY720 in the treatment of multiple sclerosis. S1P acts as an “extracellular siren song,” its high plasma concentrations stimulating pathogenic lymphocyte migration and promoting their egress from lymph nodes to blood vessels (Bandhuvula and Saba, 2007). FTY720, and more precisely its phosphorylated form, is a functional antagonist of almost all S1PRs, excepting S1P2, and prevents S1P activation of S1P1 by binding and promoting its internalization and further degradation, thus decreasing S1P1 membrane levels and retaining lymphocytes in the lymph nodes (Paugh et al., 2003; Brinkmann et al., 2010; Chun and Hartung, 2010). In addition, FTY720 has been shown to inhibit *de novo* synthesis of Cer (Berdyshev et al., 2009) and histone deacetylases (Hait et al., 2014). Its efficacy in the treatment of multiple sclerosis has led to an accelerated research on its therapeutic effects in other inflammatory diseases.

Glial cells are key players in neuroinflammation and S1P induces this inflammation in the different glial types (microglia, astrocytes, and oligodendrocytes). S1P has been linked to microglial activation; S1P addition to cultured microglial cell lines increases the release of pro- inflammatory cytokines whereas microglial activation *in vivo* increases SphK1 activity (Nayak et al., 2010; Lv et al., 2016). Astrocytes, which are involved in many central nervous system pathologies, respond to and release inflammatory mediators, and an active crosstalk exists between S1P and these mediators; S1P induces astrogliosis, whereas IL-1 induces SphK1 expression (Sorensen et al., 2003; Paugh et al., 2009). S1P1 expression in astrocytes is critical in the development of animal models of multiple sclerosis, as shown by the protective effect of FTY720 (Choi et al., 2011).

Inflammation, angiogenesis, proliferation and migration are common features in most retinopathies; hence, S1P signaling of these processes makes it an ideal candidate to regulate the onset and progression of these diseases. We will here discuss current knowledge regarding the role of S1P in eye and retina development and during retina pathological disorders, particularly the opposing functions of S1P in both neuronal survival and inflammation and fibrosis in the retina.

### S1P, Dr. Jekill or Mr. Hyde in the Retina?

Numerous findings during the last decade have shed new light on S1P functions in the retina (Table 2). SphK1 is expressed in photoreceptors whereas SphK1 and SphK2 are expressed both in ARPE19 and in human fetal RPE cells, implying these cells have the molecular machinery required for S1P synthesis (Abrahan et al., 2010; Zhu et al., 2010). A recent work has dissected the distribution of SphKs and S1PRs in mouse retina (Porter et al., 2018). *SphK1* is low in most mouse ocular tissues and highest in the retina and optic nerve, whereas higher levels of *SphK2* are observed in all mouse eye tissues. Interestingly, both SphK2 and SphK1 increase in the retina during early development, peaking at adulthood. *S1p1* and *S1p3* are expressed in the retina, while expression of *S1p2* and *S1p5* is minimal; S1P3 expression remains high and constant during retina development, whereas S1P1 increases gradually. Photoreceptors express both S1P1 and S1P3; S1P1 is highly expressed in RPE cells while S1P3 is localized in ganglion cells (Porter et al., 2018). Müller glial cells also show expression of SphK1 and S1P3 (Simón et al., 2015). Hence, the enzymes and receptors that take part in S1P signaling pathway are present in most retina cell types.

**Table 2:** Functions of Sphingosine-1-phosphate and Ceramide-1-phosphate in the retina.

Sphingolipid	Retinal Process
Sphingosine-1- phosphate (S1P)	**Normal Eye development and function**
	↓ in *Spns2* expression causes abnormal eyelid fusion (Bian et al., 2018), alterations in retinal progenitors cell cycle and laminar disorganization (Fang et al., 2018)
	↑S1P promotes proliferation of retinal progenitors and their later differentiation into photoreceptors (Miranda et al., 2009)
	↑ S1P promotes Ca^2+^ signaling in amacrine cells in the inner retina via S1P1 and S1P3 (Crousillac et al., 2009)
	**Protection against Retinal injuries**
	↑ S1P through S1P1 promotes neuronal survival and axonal sprouting after optic nerve damage (Joly and Pernet, 2016)
	FTY720 has neuroprotective effects in rat models of glaucoma (You et al., 2014)
	↓ S1P abolishes DHA–mediated protection of cultured photoreceptors from oxidative stress (Abrahan et al., 2010)
	Light- stressed retinas ↑ expression of SphK1, S1P2 and S1P3 (Porter et al., 2018)
	**Eye inflammation**
	↑ S1P promotes retinal angiogenesis and neovascularization (Eresch et al., 2018)
	↑ S1P increases IL-8 secretion in RPE (Qiao et al., 2012)
	TNF-α promotes ↑ S1P and IL-8 and IL-6 production (Qiao et al., 2012)
	↑ S1P promotes VEGF and HIF-1α production in RPE (Terao et al., 2017)
	↑ levels of S1P1 and S1P lyase in vitreous of patients with proliferative diabetic retinopathy (Abu El-Asrar et al., 2014)
	**Retinal fibrosis**
	S1P promotes proliferation, myofibroblast transformation and formation of pro- fibrotic proteins in RPE cells (Swaney et al., 2008)
	S1P promotes glial migration (Simón et al., 2015)
	Blockade of S1P with monoclonal antibodies reduces angiogenesis and sub-retinal fibrosis (Caballero et al., 2009) and prevents excessive scarring in animal models of glaucoma (Lukowski et al., 2013)

Cer-1- phosphate (C1P)	**Signaling in photoreceptors**
	C1P regulates lipid metabolism in photoreceptor outer segments (Pasquaré and Giusto, 2008; Pasquaré et al., 2008)
	C1P increases the proliferation of photoreceptor progenitors in retina neuronal cultures (Miranda et al., 2011)
	C1P promotes differentiation and prevents apoptosis in cultured rat retina photoreceptors (Miranda et al., 2011)
	↑ C1P levels in uveitis (Wang et al., 2018)
	**Phototransduction**
	CerK regulation of Cer levels required for phospholipase C activity and PIP2 production during phototransduction (Dasgupta et al., 2009)


#### S1P in Eye Development and Neuronal Survival

Sphingosine-1-phosphate has essential functions during normal eye development. Mutations in *Spns2* cause the abnormal fusion of eyelids during rat embryogenesis, which can be reversed by treatment with S1P (Bian et al., 2018). Functional SPNS2 is crucial for retinal morphogenesis, since dysfunctional SPNS2 causes delayed cell-cycle exit of retinal progenitors and retinal laminar disorganization (Fang et al., 2018). A role for S1P signaling in axon guidance has also been reported; S1P promotes repulsive turning and collapse of growth cones from ganglion cell axons in the Xenopus retina, through activation of the S1P5 and RhoA whereas loss of S1P function results in target recognition errors (Strochlic et al., 2008). S1P is also involved in signaling in inner retina cells; S1P increases cytosolic Ca^2+^ levels in cultured amacrine cells, through the activation of S1P1 and S1P3 (Crousillac et al., 2009).

Sphingosine-1-phosphate displays a vital function in preventing neuronal death during retinal injuries. S1P1 contributes to survival and axonal sprout of injured retinal ganglion cells after damage to the optic nerve (Joly and Pernet, 2016) and FTY720 has neuroprotective effects in experimental glaucoma in rats (You et al., 2014). Moreover, the expression of SphK1, S1P2, and S1P3 immediately increases in light-stressed retinas, with S1PRs localizing to the pyknotic nuclei, suggesting the up-regulation of a cytoprotective S1P signaling to counteract the onset of apoptosis (Porter et al., 2018).

#### S1P as a Mediator in Photoreceptor Survival and Differentiation

Sphingosine-1-phosphate emerges as a pleiotropic mediator for development and survival of photoreceptors. Our group established that 1 μM S1P promotes the proliferation of retinal progenitors and their later differentiation into photoreceptors; S1P increases the expression of specific photoreceptor proteins, and advances the development of rudimentary OS (Miranda et al., 2009). We also evidenced that S1P is a key mediator in photoreceptor survival, preventing photoreceptor death during development *in vitro* and when exposed to oxidative stress (Miranda et al., 2009; Rotstein et al., 2010).

An increase in S1P intracellular levels protects 661W cells from oxidative stress; exogenous addition of S1P or inhibiting S1P lyase with THI preserves viability in these cells when treated with H_2_O_2_, by decreasing Cer levels, activating the Nrf-regulated antioxidant pathway and increasing the Bcl-2/Bax ratio (Fabiani et al., 2017). Further evidence supports the proposal that S1P is an intracellular second messenger, the synthesis of which is promoted by photoreceptor trophic factors to exert its actions. GDNF promotes the proliferation of photoreceptor progenitors (Politi et al., 2001; Insua et al., 2003), as S1P does, and inhibiting SphK1 with DHS blocks GDNF mitogenic effect (Miranda et al., 2009). Inhibition of SphK1 abolishes DHA neuroprotection of photoreceptors during oxidative stress-induced apoptosis and DHA enhancement of photoreceptor differentiation (Miranda et al., 2009; Abrahan et al., 2010). Cytokines and growth factors, such as Transforming growth factor β and Nerve growth factor (NGF), enhance the expression of SphK1 and promote its rapid translocation and activation to increase the synthesis of S1P, which then acts as a second messenger to exert their effects (Toman et al., 2004; Yamanaka et al., 2004; Wattenberg et al., 2006). Noteworthy, both GDNF and DHA upregulate the expression of SphK1 and promote its translocation to the plasma membrane (Abrahan et al., 2010). Thus, GDNF and DHA might elicit their biological effects on photoreceptors by promoting SphK1 activity and increasing the synthesis of S1P, which then acts as a second messenger, essential for proper development and survival of photoreceptors. As described above, DHA prevents photoreceptor death by promoting Cer glucosylation (German et al., 2006). As a whole, these findings highlight the importance of the “sphingolipid rheostat” and of manipulating sphingolipid metabolism to promote photoreceptor survival in retinal degenerations (Figure 4).

#### S1P Signaling in Retina Inflammation and Neovascularization

Sphingosine-1-phosphate is a potent mediator in the modulation of inflammatory responses and angiogenesis. It is crucial in the regulation of lymphocyte traffic, an essential step in the pathology of inflammation (Aarthi et al., 2011; Aoki et al., 2016). Several cytokines and chemokines, including TNF-α and interleukin 1-beta (IL1-β), activate SphK1 to produce S1P, which later induces cyclo-oxygenase 2 activity (Snider et al., 2010).

Collective evidence supports the relevance of S1P in retina inflammatory and vascular diseases. Abnormal retina blood vessel growth and macular edema are major complications leading to vision loss in diabetes retinopathy and S1P emerges as a key signal in the induction of neovascularization and in the preservation of retina endothelial barrier integrity (Allende and Proia, 2002; McGuire et al., 2011). In transgenic mice that overexpress SphK2, higher retinal S1P concentration accelerates retinal angiogenesis and increases neovascularization (Eresch et al., 2018). In turn, downregulation of S1P signaling with anti-S1P monoclonal antibodies and in S1P2 null mice in an ischemia-driven retinopathy markedly reduces pathologic neovascularization (Skoura et al., 2007; Caballero et al., 2009). RPE cells express the five S1PRs, and exogenous S1P induces the secretion of Interleukin-8 (IL-8); interestingly, supplementation with TNF-α enhances S1P3 expression, with a subsequent increase in S1P-induced IL-8 and IL-6 production (Zhu et al., 2010; Qiao et al., 2012). S1P also promotes the expression of vascular endothelial growth factor (VEGF) and hypoxia inducible factor-1α in RPE cells, which are crucial angiogenic factors (Terao et al., 2017). A significant increase in the expression of S1P1 and S1P lyase is detected in the vitreous of patients with proliferative diabetic retinopathy (Abu El-Asrar et al., 2014) whereas S1P signaling through S1P1/S1P3 promotes angiogenesis *in vitro*, participating in the changes affecting pericytes and endothelial cells (Durham et al., 2015). Hence, S1P signaling through S1PRs is a driving force in the onset and progression of inflammatory and angiogenic responses in retina inflammatory diseases (Figure 3).

#### S1P and Retinal Fibrosis

Fibrosis is a pathological process characterized by the deregulation of production and deposition of extracellular matrix components, leading to the destruction of normal tissue function and architecture (Wynn, 2007). Initiated by an acute injury or a vascular damage that leads to the recruitment of inflammatory cells, subsequent secretion of pro-inflammatory cytokines, as tumor growth factor (TGF)-β, platelet derived growth factor (PDGF), connective tissue growth factor, IL-3 and also S1P conducts to the excessive production and deposition of extracellular matrix components (Kisseleva and Brenner, 2008). Although the initial damage promotes a physiological fibrotic response to accomplish tissue repair, the chronic exposure to irritation and/ or inflammation leads to the formation of a fibrotic scar that impairs functionality. Many reports establish the role of S1P in fibrotic disorders in lung (Kono et al., 2007; Milara et al., 2012), kidney (Geoffroy et al., 2005; Awad et al., 2011), liver (Davaille et al., 2002; Li et al., 2011; Liu et al., 2011) and heart (Gellings Lowe et al., 2009; Pchejetski et al., 2012). However, S1P involvement in retinal fibrosis is poorly understood.

Many retinal diseases, including AMD, diabetic retinopathy and proliferative vitreoretinopathy, have a common underlying etiology of pathological scar tissue production and the role of TGF-β and PDGF in the development of eye fibrosis is well-known (Connor et al., 1989; Gamulescu et al., 2006; Saika, 2006; Lei et al., 2007). Recent data indicate a crosstalk between S1P and TGF-β in human corneas and orbital connective tissues and suggest that S1P anti- or pro-fibrotic functions in the eye are tissue-specific (Ko et al., 2017; Nicholas et al., 2017).

The available evidence suggests that S1P is involved in retina fibrotic disorders. A monoclonal anti-S1P strategy reduces sub-retinal fibrosis in a mouse model of choroidal neovascularization and prevents the formation of excessive scarring after surgery in animal models of glaucoma (Caballero et al., 2009; Lukowski et al., 2013). Müller glial cells and RPE cells, the two main cell types that support normal retinal function, have protagonic roles in the development of retinal fibrosis (Saika et al., 2008; Bringmann and Wiedemann, 2009). S1P promotes proliferation, myofibroblast transformation, collagen production and pro-fibrotic protein expression in human RPE cells (Swaney et al., 2008). We have shown that S1P has a key role in the regulation of Müller glial cell motility; 5 μM S1P promotes the migration of cultured rat Müller glial cells through activation of S1P3 while inhibiting S1P synthesis using SphK inhibitor 2 (SphKI 2) completely blocks this migration (Simón et al., 2015). We have proposed that Müller glial cells release S1P, which signals through S1P3 and activates the PI3K and the ERK/MAPK pathway to enhance migration (Simón et al., 2015). Taking into account that the deregulation of glial migration is involved in proliferative retinopathies, the S1P/SphK1/S1P3 axis emerges as a key target for controlling these diseases.

The above findings position S1P as a real Jekill and Hyde in the retina. S1P is indispensable for establishing retina structure, regulating proliferation of retinal progenitors and their later laminar distribution in the retina (Miranda et al., 2009; Fang et al., 2018). It is also a pro-survival factor for neurons, promoting their differentiation, preserving normal functioning and granting neuroprotection (Crousillac et al., 2009; Miranda et al., 2009; Abrahan et al., 2010; Joly and Pernet, 2016; Porter et al., 2018). On the other hand, S1P unleashes threatening processes in Müller glial cells and RPE cells, such as secretion of pro- inflammatory cytokines, proliferation, migration and transdifferentiation that promote and/or enhance inflammation and fibrosis (Swaney et al., 2008; Qiao et al., 2012; Simón et al., 2015; Terao et al., 2017). Thus, S1P facilitates the formation of gliotic scars that alter retinal structure and enhance visual dysfunction instead of preventing it.

The central role of S1P in the regulation of the crucial cellular processes that are altered in retinal pathologies turns it into an exceptional therapeutic target for treating these diseases. In order to accomplish this goal, several questions remain to be answered. How to take advantage of S1P protective role on photoreceptors and simultaneously elude the potentially risks for visual function derived from its effects on glial and epithelial cells? The S1P concentration might provide a clue, since the concentration that protects photoreceptors is 5-fold lower than that enhancing glial migration; promoting a controlled intracellular synthesis of S1P and preventing its release might preclude S1P deleterious effects.

Whether S1P dual roles depend on the signaling pathways it activates in each cell type and whether they respond to the microenvironment and/ or the type of injury cells are exposed to are pending questions as well. An attractive hypothesis for the dual role of S1P in retina degeneration is its function as a DAMP, signals that are exposed or released from stressed cells and recognized by and mobilize the immune system. S1P and Cer have been proposed as DAMPs in cancer cells; treating squamous carcinoma cells by photodynamic therapy results in Cer and S1P increases on the cell surface and in S1P release, and both sphingolipids trigger NFκB signaling in macrophages in co-culture (Korbelik et al., 2014). The ubiquitous expression of S1PRs in macrophages supports this proposal (Fischer et al., 2007). Recent research has shown that apoptotic cells release exosome-like vesicles, the biogenesis of which depends on S1P/S1PRs signaling; these vesicles have S1P, S1P1, and S1P3 and induce genes of proinflammatory cytokines and chemokines (Park et al., 2018), highlighting a new pathway for S1P in the pathogenesis of inflammatory diseases. Microglia, as resident retinal macrophages, has a detrimental role and contributes to retinal degeneration; microglia release of harmful factors contributes to Müller glial cell reactivity in *rd10* mice (Peng et al., 2014; Zhao et al., 2015). DAMPs have dual roles in the retina; e.g., fractalkine, which has been reported as a DAMP, is neuroprotective in *rd10* retinas by signaling through its CX3CR1 receptor, preventing microglia-induced damage (Roche et al., 2016). Stressed photoreceptors release DAMPs early in degeneration to induce neuroprotective responses in retinal glial cells by interacting with receptors as Toll-like receptor 2, a DAMP receptor that increases in Müller glial cells during degeneration (Hooper et al., 2018). S1P promotes migration in Müller glial cells and RPE cells (Swaney et al., 2008; Simón et al., 2015), which might release it to the retina milieu to act as an autocrine signal and promote a fibrotic process and/or to act as a paracrine signal in photoreceptors, promoting neuroprotection. Whether S1P is a DAMP released by retinal cells to either promote inflammation or neuroprotection is an exciting question to be explored in the sphingolipid field.

Finding answers to these questions would provide crucial understanding of the puzzling behavior of S1P and new insight to design effective therapeutic for the treatment of retinopathies.

## Searching for Ceramide-1-Phosphate Functions in the Retina

### C1P Synthesis and Functions

Ceramide-1-phosphate is a later incorporation to the sphingolipid family, now established as a pleiotropic bioactive sphingolipid with multiple cellular roles. CerK was first identified in brain synaptic vesicles and shown to synthesize a molecule identified as C1P (Bajjalieh et al., 1989). C1P existence and its synthesis from Cer were then demonstrated in the human pro-myelocytic leukemia cell line HL-60 (Dressler et al., 1992). Cer phosphorylation by CerK is the only mechanism for C1P generation established in mammals, although the presence of a small pool of C1P in CerK-/CerK- mutants suggests an alternative biosynthetic mechanism must exist (Bornancin, 2011). C1P is synthesized in the *trans* Golgi and a specific C1P transfer protein transports it to the plasma membrane, where it can be released for autocrine or paracrine signaling (Lamour et al., 2007; Simanshu et al., 2013). CerK is present in the brain and brain synaptic vesicles (Bajjalieh et al., 1989; Bajjalieh and Batchelor, 2000; Hannun et al., 2001) whereas cerebellar granule cells generate C1P from newly synthesized Cer, formed through SM degradation and Sph recycling (Riboni et al., 2002).

First thought as an intermediate in the complex sphingolipid metabolism, C1P functions began to be unraveled when it was shown to promote proliferation, in fibroblasts and macrophages (Gomez-Muñoz et al., 1995, 1997; Gangoiti et al., 2008), myoblasts and cancer cells (Mitra et al., 2007; Gangoiti et al., 2008). C1P is now known to be antiapoptotic (Gómez-Muñoz et al., 2004; Granado et al., 2009), to promote cell migration in different cell types (Granado et al., 2009; Arana et al., 2012), and to regulate the production of TNF-α (Lamour et al., 2011). Although mostly known for its pro-inflammatory actions, both C1P and CerK have been shown to exert either pro- or anti-inflammatory actions depending on the cell type (Presa et al., 2016). C1P has been proposed to be relevant for cancer progression, promoting tumor cell survival, growth and migration (Mitra et al., 2007; Rivera et al., 2016).

Ceramide-1-phosphate is also an intracellular and an extracellular messenger. In most cases, it acts as an intracellular signal, but it can be released to the extracellular milieu in specific circumstances or upon cell damage (Boath et al., 2008; Kim et al., 2012; Schneider et al., 2013). The Gomez-Muñoz laboratory demonstrated that exogenous C1P stimulates migration of macrophages independently of intracellular C1P synthesis by activating a G-protein coupled membrane receptor for C1P, partially identified and different from S1PRs (Granado et al., 2009). In contrast, C1P mitogenic effect on macrophages depends on its action as an intracellular messenger, as C1P effect can be mimicked by a photolabile caged-C1P analog, which activates cPLA2, PKCα and NADPH oxidase, generating ROS that promote proliferation (Arana et al., 2012).

Ceramide-1-phosphate activates multiple signaling pathways. To enhance proliferation, C1P activates the PI3K and the ERK/MAPK pathways, Jun N terminal kinase (JNK), nuclear factor NF-κB (NF-κB) and glucogen synthase kinase 3 (GSK3) (Gangoiti et al., 2010). C1P promotes survival of macrophages in the absence of trophic factors by inhibiting caspases and blocking Cer synthesis (Gómez-Muñoz et al., 2004; Granado et al., 2009). Activation of PI3K, NF-κB, increases in Bcl-2, decreases in Bax levels and inhibition of caspases 9 and 3 also participate in its survival effects (Gómez-Muñoz et al., 2005; Gomez-Muñoz, 2018). The findings by the Chalfant laboratory that C1P is a direct activator of cPLA2 provided the link between this sphingolipid and inflammation (Pettus et al., 2004). C1P interacts with cPLA2, promoting its translocation and association to membranes, and its subsequent activation, producing inflammatory mediators such as eicosanoids (Lamour et al., 2009; Hoeferlin et al., 2013; Simanshu et al., 2013).

Mounting evidence points to different roles for C1P in the nervous system. A 5-fold increase in C1P levels in the subventricular zone in Huntington disease patients has been reported, with no associated changes in Cer levels; this increase might represent a response to the chronic brain damage in these patients and might either reflect an attempt at neuroprotection and enhanced neurogenesis or contribute to neuroinflammation and neurodegeneration (Hunter et al., 2018). C1P has neuroprotective effects in the nervous system. Upregulation of CerK by Peroxisome proliferator-activated receptors protects neurons and astrocytes against Cer-induced death (Aleshin and Reiser, 2014) and promotes survival of cochlear hair cells during ototoxicity (Tabuchi and Hara, 2018). C1P might also be involved in neurotransmitter release; C1P promotes dopamine release from PC12 cells (Jeon et al., 2005), which is consistent with the ability of C1P to promote phagolysosome formation and with CerK presence in synaptic vesicles (Bajjalieh et al., 1989; Hinkovska-Galcheva et al., 1998).

### C1P in the Retina

Little is known regarding C1P and CerK roles in the eye (Table 2). Pioneer work from Acharya’s group in *Drosophila* eye revealed an indirect effect of *Drosophila* ceramide kinase (DCerK) in phototransduction. DCerK is an integral membrane protein that phosphorylates Cer, thus decreasing its levels (Dasgupta et al., 2009). DCerK mutants show severe photoreceptor degeneration and do not respond to light. The absence of DCerK increases Cer levels, and leads to proteolysis of NORPA, a critical effector of phototransduction. The degradation of this phospholipase C homolog leads to a consequent loss of activity, and failure in light signal transduction (Dasgupta et al., 2009). Thus, modulation of Cer levels by DCerK is essential for phototransduction. Noteworthy, C1P is involved in light-dependent regulation of other enzymes of lipid metabolism, such as diacylglycerol lipase and lipid phosphatases, in photoreceptor rod OS (Pasquaré and Giusto, 2008; Pasquaré et al., 2008), suggesting its role in phototransduction might also extend to the mammalian retina.

The finding that mutations in a CerKL gene are associated to a variant of autosomal recessive retinitis pigmentosa (Tuson et al., 2004), and to Cone-rod dystrophy (Littink et al., 2010; Birtel et al., 2018) brought huge expectations to the field, as it associated sphingolipid metabolism and retinal degeneration. CerKL has been further connected with retinitis pigmentosa in numerous studies (Auslender et al., 2007; Avila-Fernandez et al., 2008; Avela et al., 2018). However, the physiological functions of CerKL and its contribution to degeneration are still unclear. CerKL has no kinase activity neither on lipids nor on proteins in spite of having a diacylglycerol kinase domain (Tuson et al., 2004, 2009; Bornancin et al., 2005) and it interacts with several calcium sensor proteins in the retina (Nevet et al., 2012). A targeted deletion of CerKL in mouse leads to a mild retinal phenotype, with increased gliosis and mild functional alterations of the ganglion cell layer but no gross morphological alterations (Garanto et al., 2012). In contrast, CerKL knockdown causes retinal degeneration in zebrafish, with failure in the development of photoreceptor OS and increased apoptosis (Riera et al., 2013). CerKL might have antioxidant functions in the retina. Its overexpression protects retinal cells from oxidative stress-induced death, whereas its downregulation renders cells sensitive to this damage; CerKL deficiency causes zebrafish retinal degeneration and photoreceptor apoptosis through increased oxidative damage (Tuson et al., 2009; Li et al., 2014). CerKL is prominently localized in RPE cells, ganglion cells, inner nuclear layer and photoreceptor inner segments and its expression increases in light-stressed retinas (Mandal et al., 2013). An alteration in OS phagocytosis in a zebrafish CerKL knockout suggests a role for CerKL in RPE cell phagocytosis, leading to rod-cone dystrophy (Yu et al., 2017). Recent miRNA analysis of RPE cells exposed to oxidative stress shows that CerKL is a target of five altered miRNA (Donato et al., 2018). Existing evidence thus points to a role for CerKL in retina protection from oxidative stress-induced damage, and not related to Cer phosphorylation.

Ceramide kinase is ubiquitously and highly expressed in the retina (Mandal et al., 2013). CerK^-^/- retinas show decreased C1P and increased Cer; on the contrary, CerKL-/- retinas have normal C1P and Cer levels, supporting CerK as the major and probably unique responsible for C1P synthesis in the retina (Graf et al., 2008). RPE cells express CerK, among other enzymes involved in sphingolipid metabolism (Zhu et al., 2010) and no C1P is detected in these cells in CerK^-^/- retinas (Graf et al., 2008). Our group has shown that exogenous C1P promotes the proliferation of cultured photoreceptor progenitors and advances their differentiation as photoreceptors, enhancing the expression of opsin and peripherin and promoting the formation of rudimentary OS (Miranda et al., 2011). Notably, overexpression of CerK inhibits all-trans retinoic acid (ATRA)-induced differentiation of a human neuroblastoma cell line, SH-SY5Y (Murakami et al., 2010), suggesting that the effect of C1P on differentiation might be cell or context dependent. C1P also stimulates the survival of cultured photoreceptors, preventing their degeneration; this protection involves the preservation of mitochondrial potential, suggesting that C1P activates pathways upstream of mitochondrial depolarization (Miranda et al., 2011). Consistently, whereas a pan-caspase inhibitor decreases photoreceptor death in controls, it promotes no further reduction in photoreceptor death in C1P-supplemented cultures, suggesting C1P might prevent caspase activation (Miranda et al., 2011). Inhibiting the *de novo* synthesis of Cer prevents photoreceptor cell death in culture (German et al., 2006). Since C1P prevents macrophage apoptosis by impeding Cer accumulation, either by inhibiting its *de novo* synthesis or acid SMase activity (Gómez-Muñoz et al., 2004; Granado et al., 2009), C1P might also block Cer synthesis in photoreceptors, thus promoting their survival (Figure 3, 4).

Higher levels of C12-, C16- and C24-C1P, and of C16- and C18-Cer have been found in retinas in a rat model of endotoxin-induced uveitis, together with increases in TNF-α and IL-6 levels, suggesting these sphingolipids might participate in the inflammation process (Wang and Bieberich, 2018). Ongoing work in our laboratory suggests that C1P might promote the migration of retina Müller glial cells *in vitro*, which, together with glial proliferation, contributes to the progression of proliferative retinopathies (Vera et al., unpublished data).

Ceramide-1-phosphate functions in the retina are just starting to be uncovered. Existing evidence points to a crucial role of C1P in photoreceptor functionality, promoting their proliferation, survival and advancing differentiation. It might also be involved in modulation of phototransduction. Its contribution to inflammatory and proliferative pathologies of the retina has to be further explored.

## Dihydroceramide, a Newcomer Player in Retina Cell Death?

Dihydroceramide, the immediate precursor of Cer in the *de novo* synthetic pathway, had long been considered as an inactive sphingolipid, since its exogenous addition had little effect on cell death (Ahn and Schroeder, 2010). However, cumulative evidence during the last decade supports a signaling role for endogenous DHCer and for other dihydrosphingolipids as well. Its accumulation has been implicated in autophagy regulation. Noteworthy, DHCer increase has been shown to induce cytotoxic autophagy in cancer cells and an impairment of autophagic flux and increase in fibrosis markers in liver cells (Hernández-Tiedra et al., 2016; Lee et al., 2017). DHCer also triggers cell cycle arrest and cell death (reviewed in Siddique et al., 2015). Therefore, it is now thought that DHCer is the principal Cer-mediated autophagy regulator (Siddique et al., 2015; Hannun and Obeid, 2018).

In the eye, early work showed distinct effects between Cer and DHCer. Cer induces cell death in cultured rat photoreceptors and amacrine neurons, in human RPE cells, in human corneal stromal fibroblasts and in lens epithelial cells, whereas addition of DHCer has no deleterious effect in these cell types (Barak et al., 2001; German et al., 2006; Samadi, 2007; Rizvi et al., 2011). However, recent evidence suggests a noxious role for DHCer in the eye. DHCer content increases during aging in human lens and might be related to changes in the permeability barrier properties (Deeley et al., 2010). Both Cer and DHCer content increase in light-induced photoreceptor degeneration in albino rats, and preventing these increases protects retinal function and structure (Chen et al., 2013; Huang et al., 2014). DHCer desaturase catalyzes the conversion of DHCer to Cer and its modulation is crucial for the signaling pathways regulated by both sphingolipids. Loss of DHCer desaturase in *Drosophila* eye leads to DHCer accumulation, increased ROS production and loss of photoreceptor functionality and viability; in turn, blocking DHCer synthesis preserves their viability (Jung et al., 2017). Further work will establish whether DHCer is a member of the club of deadly sphingolipid messengers and whether it contributes to retinal pathologies.

### Unraveling the Sphingolipid Network in the Retina

Sphingolipids emerge as crucial mediators in the activation of numerous pathways that might either activate neuroprotective mechanisms or whose dysregulation might lead to retina dysfunction. Cer stands out as a key messenger in the onset of degeneration in photoreceptors induced by oxidative stress and in numerous models of retina neurodegeneration; its increase through *de novo* synthesis or by SM breakdown activates different downstream pathways leading to cell death. Cer buildup, due to an activation or increased expression of SMases is also crucial in the onset of RPE cell death and inflammation. Preventing Cer increase through the inhibition or modulation of different enzymes of its intricate metabolism has been shown to effectively prevent photoreceptor and RPE cell death, both *in vivo* and *in vitro*, and preserve visual function. These findings underscore both the relevance of imbalances in Cer levels as inducers of RPE and photoreceptor cell death and the significance of modulation of the sphingolipid metabolism as a therapeutic strategy for the treatment of retina neurodegenerations. Having an integral picture of the functions of Cer in the retina and of the effects of preventing its accretion demands establishing the processes regulated by Cer in glial cells and its potential role in their gliotic response. Several questions remain to be answered in order to accomplish the fine tuning of the highly interconnected sphingolipid metabolism, which is crucial for the development of new treatments for retinal pathologies. The complexity of this metabolism makes its modulation a powerful approach for preserving vision function but might also be an Achilles’ heel, jeopardizing strategies based on the inhibition of one of the metabolic pathways leading to Cer synthesis. Being Cer the central molecule in sphingolipid metabolism, the effects of blocking a single pathway established as crucial in generating Cer accumulation in a particular pathological circumstance should be explored. This inhibition might efficiently reduce cell death but impair other key, Cer-dependent, retinal processes or affect them indirectly, through the increase in bioactive sphingolipid metabolites. As blocking a certain biosynthetic pathway for Cer has been shown to provide a transient protection, it is critical to explore whether altering the flux through the network activates alternative Cer biosynthetic pathways and/or leads to the accumulation of bioactive sphingolipids, such as Sph or DHCer, which might trigger cell death. Further research aimed at understanding these balances and connections in a comprehensive molecular way might contribute to devising successful treatments based in new or already established drugs or suggest the necessity of combining different drugs in order to simultaneously modulate these metabolic processes.

Modulation of the sphingolipid rheostat might provide clues for preventing the death of photoreceptors (Figure 4). Nevertheless, S1P functions remain enigmatic. S1P, either exogenous or generated intracellularly, promotes survival of these cells; notably, photoreceptor trophic factors enhance S1P synthesis to exert their functions. A pending question is determining whether increasing S1P intracellular levels, either by promoting its synthesis and/or or blocking its degradation can prevent degeneration of photoreceptors and preserve their functionality in diseased retinas. Even if this is the case, taking advantage of S1P neuroprotective effect on photoreceptors still requires uncovering the clues to prevent or overcome S1P deleterious effects on other retinal cells, as glial, epithelial and endothelial cells, that may turn on fibrosis, neovascularization and inflammation. Similarly, the actions of S1P and Cer are critical for the pathogeneses of proliferative diseases, such as diabetic retinopathy and AMD. Both sphingolipids drive forward the crucial events leading to the progression of these diseases, with S1P inducing fibrosis and pro-inflammatory changes and Cer triggering cell death. Given the cross-conversions of sphingolipids, it is necessary to find out how to simultaneously balance their levels to avoid the onset of lethal processes in the retina. As stated above, this requires unraveling the intricate network of metabolic and signaling pathways to obtain the cues for refining the adjustment of the sphingolipid network. At least part of these cues might lie in the differential cellular expression of S1PRs and/or the molecular pathways activated by S1P. Uncovering the myriad of potential combinations of S1PRs and downstream signaling pathways, either activatory or inhibitory, might explain the variety of the responses triggered by S1P in different retinal cell types. In order to make use of the S1P axis as a tool for the treatment of retinal diseases, the precise mechanisms by which S1P achieves its effects should be clarified. Current evidence suggests that the expression pattern of S1PRs in the retina is modified in particular pathologies. Further studies are now required to establish whether photoreceptors, glial and epithelial cells differ in their S1PR expression pattern, and if and how are these patterns differentially modulated in each cell type by the environmental context, i.e., healthy or diseased retina and by extracellular cues, such as starvation, light damage, or oxidative stress. In addition, it is essential to understand the roles of S1P, and also establish those of C1P, in controlling neovascularization and fibrotic changes and whether they exhibit pro or anti-inflammatory actions in different pathological environments. Advancing our understanding of the multiple roles of sphingolipids and of the fine detail and regulation of their complex metabolic network will contribute to achieve much-needed therapies for retina pathologies.

## Author Contributions

MS wrote and edited the manuscript and contributed to the design of the figures. FPS wrote and edited the manuscript and contributed to the design of the figures. MV wrote the manuscript. NR designed the manuscript and figures and wrote and edited the manuscript.

## Conflict of Interest Statement

The authors declare that the research was conducted in the absence of any commercial or financial relationships that could be construed as a potential conflict of interest.

## Abbreviations

AMD: age related macular degeneration
C1P: ceramide-1-phosphate
Cer: ceramide
CerK: ceramide kinase
CerKL: Cer kinase-like
CerS: Cer synthase
CERT: Cer transport protein
DAMP: damage associated molecular pattern
DHA: docosahexaenoic acid
DHCer: dihydroceramide
ER: endoplasmic reticulum
GDNF: glial derived neurotrophic factor
GlucoCer: glucosylceramide
OS: outer segments
PARP1: poly ADP-ribose-polymerase 1
PUFA: polyunsaturated fatty acids
ROS: reactive oxygen species
RPE: retinal pigment epithelium
S1P: sphingosine-1-phosphate
S1PR: S1P receptor
SM: sphingomyelin
SMase: sphingomyelinase
Sph: sphingosine
SphK: sphingosine kinase
SPP: S1P phosphatases
SPT: serine palmitoyltransferase
TNF: tumor necrosis factor
VLCPUFA: very long chain polyunsaturated fatty acids
